# A Quick Mind with Letters Can Be a Slow Mind with Natural Scenes: Individual Differences in Attentional Selection

**DOI:** 10.1371/journal.pone.0013562

**Published:** 2010-10-27

**Authors:** Sander Martens, Mathijs Dun, Brad Wyble, Mary C. Potter

**Affiliations:** 1 Neuroimaging Center, University of Groningen, Groningen, The Netherlands; 2 Department of Neuroscience, University Medical Center Groningen, Groningen, The Netherlands; 3 Department of Psychology, Syracuse University, Syracuse, New York, United States of America; 4 Department of Brain and Cognitive Sciences, Massachusetts Institute of Technology, Cambridge, Massachusetts, United States of America; University of Regensburg, Germany

## Abstract

**Background:**

Most people show a remarkable deficit in reporting the second of two targets (T2) when presented 200–500 ms after the first (T1), reflecting an ‘attentional blink’ (AB). However, there are large individual differences in the magnitude of the effect, with some people, referred to as ‘non-blinkers’, showing no such attentional restrictions.

**Methodology/Principal Findings:**

Here we replicate these individual differences in a task requiring identification of two letters amongst digits, and show that the observed differences in T2 performance cannot be attributed to individual differences in T1 performance. In a second experiment, the generality of the non-blinkers' superior performance was tested using a task containing novel pictures rather than alphanumeric stimuli. A substantial AB was obtained in non-blinkers that was equivalent to that of ‘blinkers’.

**Conclusion/Significance:**

The results suggest that non-blinkers employ an efficient target selection strategy that relies on well-learned alphabetic and numeric category sets.

## Introduction

People differ widely in their ability to focus attention on meaningful stimuli (e.g., a red traffic light) while ignoring irrelevant stimuli (e.g., a billboard). A suitable paradigm to study individual differences in the temporal dynamics of attentional selection is that of the attentional blink (AB) [1]. In this paradigm two targets (e.g., letters) presented in a rapid serial visual presentation (RSVP) stream of irrelevant distractors (e.g., digits) must be detected or identified. The majority of participants, which we refer to as ‘blinkers’, often fails to report the second of the two targets (T2) when it occurs 200–500 ms after the first (T1). Although this interference effect is very robust and can be obtained under a variety of stimuli and task conditions [2], the magnitude of the AB effect varies from one individual to another. Some individuals (∼5% of the population), referred to as ‘non-blinkers’, even show no visual AB whatsoever [3]. Given that the AB is widely assumed to reflect a fundamental limitation in information processing, an intriguing question is why non-blinkers do not show an AB.

Comparing these non-blinkers to strong blinkers, no differences have been found either in working memory, short-term memory, or general intelligence [4]. However, we have previously presented psychophysiological evidence showing that target processing differs in blinkers and non-blinkers [3]. EEG measurements revealed differences in parietal brain activity, suggesting that non-blinkers are quicker to consolidate the identity of targets than blinkers are. In addition, non-blinkers showed more target-related activity over the right ventrolateral prefrontal cortex (assumed to play a role in a wide range of cognitive processes, including selection of nonspatial information), whereas blinkers showed more distractor-related pre-frontal activity. This higher level of distractor-related activity in blinkers suggests that they may direct more attention to each distractor than non-blinkers do. Indeed, behavioral evidence confirmed that non-blinkers are better at ignoring distractors than blinkers are [5], [6].

Non-blinkers continue to show little or no AB when identification of targets is made more difficult, either by increasing the overall rate of stimulus presentation [3], or by specifically reducing the duration of the targets [7]. However, when stimuli are presented in the auditory modality, non-blinkers do show a substantial AB effect, suggesting that their remarkable ability to perceive two targets without exhibiting an AB might be specific for the visual modality [7].

Considering these lines of evidence, it has been suggested that non-blinkers select visual targets at an early, pre-consolidation stage, thus reducing the amount of competition with irrelevant distractors within working memory, thereby preventing the occurrence of an AB [3], [4], [5], [7], [8]. The goal of the current study was to test the generality of the non-blinkers' superior selection of visual targets. To that end, non-blinkers and blinkers were first tested in a AB task consisting of alphanumeric stimuli (Experiment 1A), followed by an AB task consisting of picture stimuli (Experiment 1B).

## Methods

### Experiment 1A

In Experiment 1A an AB task with alphanumeric stimuli required the detection and identification of two target letters presented in an RSVP stream of 16 distractor digits. Participants were tested for the presence or absence of a sizeable AB, with the purpose of forming separate groups of consistent blinkers and non-blinkers for inclusion in Experiment 1B.

#### Participants

Twenty-nine volunteers (aged 18–28, mean = 22.7) were recruited from the University of Groningen community, and were tested for the occurrence of a significant AB effect with alphanumeric stimuli. Fifteen of those individuals were recruited because they had previously shown little or no AB in AB experiments in our laboratory, and were therefore regarded as potential non-blinkers. The other fourteen participants were new, and were considered to be potential blinkers.

All participants had Dutch as their native language, normal or corrected-to-normal visual acuity and no history of neurological problems. The Neuroimaging Center Institutional Review Board approved the experimental protocol and written consent was obtained prior to the experiment. Participants received payment of € 7.

#### Stimuli and apparatus

The generation of stimuli and the collection of responses were controlled by using E-prime 1.2 software [9] running under Windows XP. Distractor stimuli consisted of digits (2 to 9) and target stimuli of uppercase consonant letters (excluding ‘Q’ and ‘Y’). All stimuli were presented in black (2 cd/m^2^) on a white background (88 cd/m^2^) presented in 12-point courier new font on a 19-inch CRT monitor with a 100-Hz refresh rate.

#### Procedure

Each trial began with a message at the bottom of the screen, prompting participants to press the space bar to initiate the trial. When the space bar was pressed the message disappeared immediately and a fixation cross appeared which remained on the screen for 100 ms, followed by the RSVP stream consisting of 18 items.

Distractors were presented for 100 ms. Each block began with a target duration of 90 ms, immediately followed by a 10-ms mask (a digit). Following Martens et al. [7], we attempted to control task difficulty, keeping mean T1 performance at approximately 85%, by manipulating the duration of the targets in the following way. After the first trial, target and mask duration were variable, with target duration ranging from 20 to 90 ms. The sum of target and mask duration was always 100 ms, thereby keeping the interval between the onset of a target and the onset of a subsequent distractor constant. After each trial a running average of T1 accuracy was calculated. Whenever mean T1 accuracy became higher than 90%, target presentation was decreased by 10 ms and mask duration was increased by 10 ms, thereby making T1 identification more difficult. When T1 mean accuracy dropped below 80%, target presentation duration was increased by 10 ms and mask duration decreased by 10 ms, thereby making T1 identification easier. T1 was always presented as the sixth item in the stream. T2 was the first, second, third, or eighth item following T1 (i.e., it was presented at lag 1, 2, 3, or 8, respectively). Thus, the stimulus onset asynchrony (SOA) between the targets randomly varied from 100, 200, 300, to 800 ms. Each SOA was presented equally often. Target letters were randomly selected with the constraint that T1 and T2 were always different letters. Digit distractors and masks were randomly selected with the constraint that no single digit was presented twice in succession. There was no inter stimulus interval between any of the stimuli.

After the presentation of the stimulus stream, participants were prompted by a message at the bottom of the screen to type the letters they had seen using the corresponding keys on the computer keyboard. Participants were instructed to take sufficient time in making their responses to ensure that typing errors were not made, and to press the space bar instead of a letter if they had not seen it. They were encouraged to type in their responses in the order in which the letters had been presented, but responses were accepted and counted correct in either order. The task consisted of one practice block of 24 trials and two testing blocks of 128 trials each, and took approximately 20 minutes to complete.

### Experiment 1B

Whereas an AB can be observed in most people, Experiment 1A replicated earlier observations that some individuals, referred to as non-blinkers, show little or no visual AB in a task requiring the identification of two target letters embedded in a stream of digit distractors [3], [4], [5], [7], [8]. To address the question of whether the same pattern of results can be found with other types of visual stimuli, selected blinkers and non-blinkers from Experiment 1A volunteered to perform an AB task containing pictures of natural scenes. Participants were required to identify two pictures (e.g., an ‘orchid’ and a ‘rose’) that belonged to a superordinate category (‘flowers’) that was specified at the start of each trial. Each picture was unique, being presented only once throughout the experiment. The picture AB task in Experiment 1B thus prevented target selection from being based on overlearned perceptual features or alphanumeric category information. If the selection strategy employed by the non-blinkers relies on overlearned perceptual information, non-blinkers should now have an AB like that of blinkers.

#### Participants

On the basis of performance in Experiment 1A, two groups of participants were formed. Following Martens, et al. [3], individual AB magnitudes were computed according to the following formula:
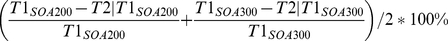



Of the 15 candidate blinkers from Experiment 1A, 12 were selected for inclusion in the blinker group of Experiment 1B (aged 18–28, mean = 22.3), showing an AB magnitude of more than 20% in the alphanumeric AB task. Eleven of the fifteen candidate non-blinkers were selected for inclusion in the non-blinker group of Experiment 1B (aged 18–27, mean = 22.3). One of the candidate blinkers, who were new participants, turned out to be a non-blinker with an AB magnitude of 7.4% and was therefore assigned to the non-blinker group. All selected individuals volunteered to participate in Experiment 1B. The twelve blinkers had a mean AB magnitude of 44.5% (range = 20.1 to 64.6%), whereas the twelve non-blinkers had a mean AB magnitude of only 6.8% (range = −.6 to 18.6%), which was significantly different according to an independent samples t-test, t(22) = 8.62, SE = 4.37, p<.001. As an alternative measure of AB magnitude, comparing T2 performance during the AB period relative to T2 performance at SOA 800 (lag 8) rather than T1 performance, also revealed a significantly smaller AB magnitude in non-blinkers (2.2%) than in blinkers (23.9%), t(22) = 8.62, SE = 5.17, p<.001. Finally, intra-individual stability of performance was checked on odd and even number trials for these participants. The Spearman-Brown prophecy coefficients were .41, .96, .94, and .79, for T1, T2|T1, AB magnitude relative to T1, and AB magnitude relative to T2 at SOA 800, respectively. While it is unclear why the intra-individual stability of T1 performance was relatively low, the other values reflect stable individual performance, similar to what was found in previous AB studies [3], [5], [7], [10].

#### Stimuli and apparatus

The picture AB task contained 640 colored images downloaded from Google Images, depicting single objects in their natural or most commonly seen setting, as well as natural scenes of everyday settings. Pictures were retouched to remove unwanted visual features or text, and resized to 300×200 pixels. The same pictures had previously been used in a similar paradigm by Potter and colleagues [11], who had observed a significant AB effect. The apparatus was the same as in Experiment 1A.

#### Procedure

The picture AB task required detection and identification of two semantically related pictures embedded within an RSVP stream of 6 non-related distractor pictures. Each trial began with a message at the bottom of the screen, prompting participants to press the space bar to initiate the trial. When the space bar was pressed the message disappeared immediately and a fixation cross appeared, which remained on the screen for 400 ms. Subsequently, the target category was displayed for 750 ms, followed by the RSVP stream consisting of 8 stimuli.

All stimuli were presented for 110 ms, and each picture was presented only once throughout the experiment. Target duration was not manipulated in the picture AB experiment. T1 was always presented as the second or third item in the stream. T2 was the first, second or fourth item following T1 (i.e., it was presented at lag 1, 2, or 4, respectively). Thus, the stimulus onset asynchrony (SOA) between the targets was 110, 220, or 440 ms. Because substantial differences existed in identification difficulty between the pictures and only a limited set of stimuli was available, a fixed randomized order was used. Only the order of trials was randomized, and the combination of SOA, T1 position, and target order assigned to a set of pictures was counterbalanced across participants. To facilitate the comparison of blinkers and non-blinkers, each participant in the blinker group was paired to a participant in the non-blinker group, such that both of them received the exact same combination of SOA, T1 position, and target order for a specific picture stream.

After the presentation of the stimulus stream, participants were asked to verbally report to the experimenter which of the presented targets they were able to identify. They were instructed to give the name of each target, not its category, and if they did not know the name, to describe the object. The answers given by the participants were typed in by the experimenter using a keyboard. The experiment consisted of a testing block containing 72 trials, preceded by a practice block containing 8 trials, and took approximately 30 min in total to complete.

Both the experimenter and a colleague who had no information regarding group membership rated the given answers as correct or incorrect. Following [11], a response was scored as correct if it was the name we gave the object or a synonym of that name. Responses that were the name of a closely related object in the same category for which the object might have been mistaken such as a papaya and mango were also counted as correct, as were responses that provided a close, correct description of the object. All other responses and omissions were scored as incorrect. Discrepancies between the scoring of the assessors were presented to a third assessor, who also had no information regarding group membership and had the final say. In this manner, a bias in scoring due to expectations of the experimenter was prevented.

## Results and Discussion

### Experiment 1A

**Figure 1:**
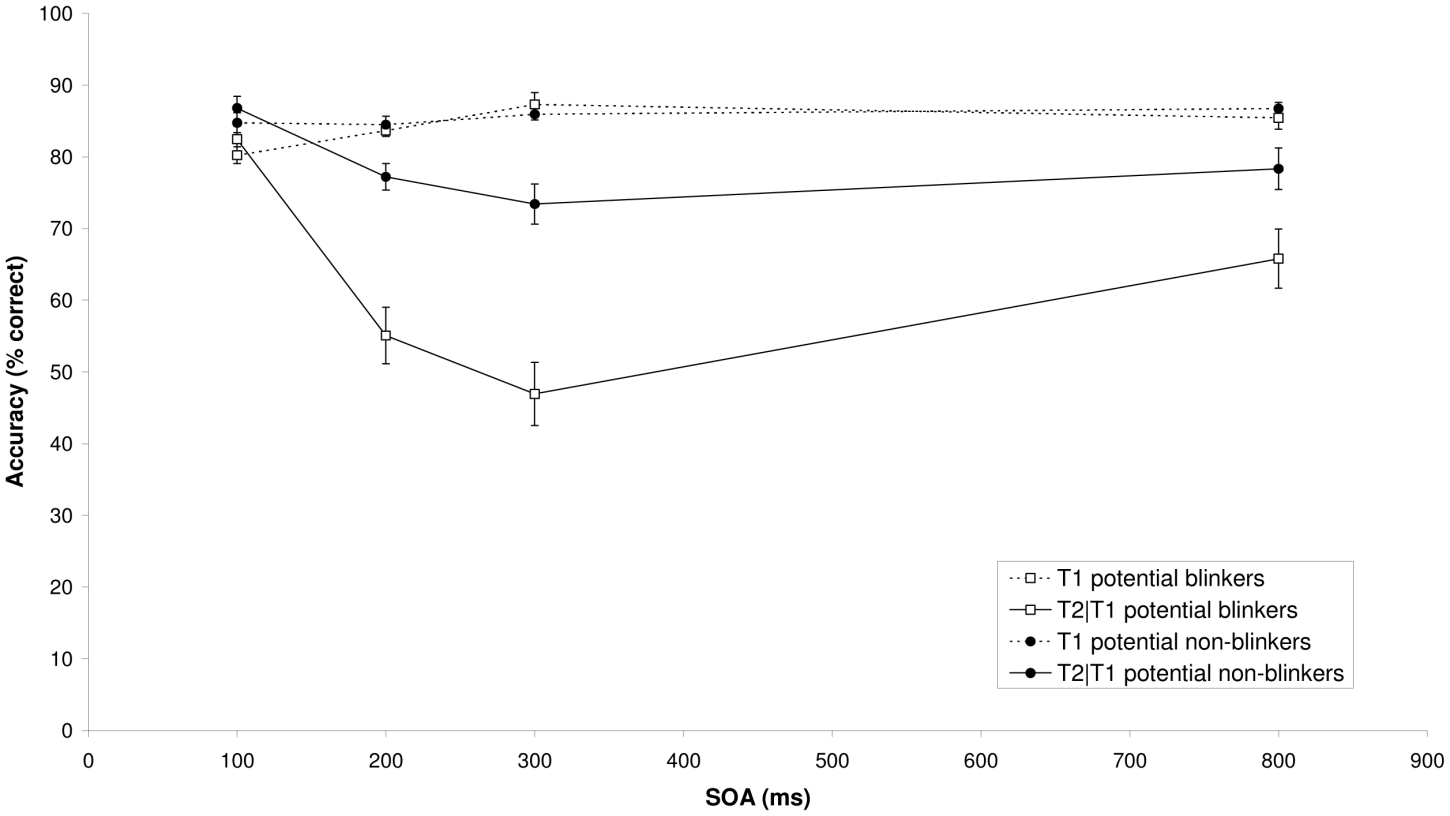
Target accuracy in Experiment 1A. Mean percentage correct report of T1 (dotted lines) and T2 given correct report of T1 (solid lines) as a function of SOA for candidate blinkers (square symbols) and non-blinkers (circle symbols) in the alphanumeric AB task of Experiment 1A.

When appropriate, Greenhouse-Geisser-corrected p values are reported. Figure 1 shows T1 identification performance as a function of the interval between the two targets (SOA) for the group of potential blinkers and the potential non-blinkers, respectively. Mean T1 performance was 85.5% for the candidate non-blinkers and 84.1% for the candidate blinkers. A mixed analysis of variance (ANOVA) of T1 performance with group (candidate non-blinkers and blinkers) as a between-subjects factor and SOA (100, 200, 300, and 800 ms) as a within-subjects factor revealed no significant effect of group (p = .18). There was a main effect of SOA, F(3, 81) = 5.41, MSE = 19.43, p = .002, η^2^_p_ = .17, reflecting T1 performance at SOA 100 (lag 1) to be somewhat lower than at the other SOAs. The Group×SOA interaction was not significant (p = .10).

Importantly, the lack of a main effect of group for T1 performance suggests that, by applying variable target and mask duration, overall task difficulty was successfully controlled. Mean target duration was significantly lower for the candidate non-blinkers (72.5 ms) than for the candidate blinkers (80.0 ms), t(27) = 2.17, SE = 3.47, p = .04. This suggests that, without variable target and mask duration, blinkers might have had more difficulty in reporting T1 than the non-blinkers. However, by keeping T1 performance comparable for both groups, any differences in AB magnitude are unlikely to be due to differences in target identification difficulty.

Figure 1 also shows T2 performance given that T1 was identified correctly, as a function of SOA for both groups. An ANOVA with group (candidate non-blinkers and blinkers) as a between-subjects factor and SOA (100, 200, 300, and 800 ms) as a within-subjects factor revealed a significant effect of group, F(1, 27) = 22.02, MSE = 352.92, p<.001, η^2^_p_ = .45, and SOA, F(3, 81) = 49.24, MSE = 64.18, p<.001, η^2^_p_ = .65. In addition, a significant Group×SOA interaction was found, F(3, 81) = 11.05, MSE = 64.18, p<.001, η^2^_p_ = .29, reflecting a clear difference in AB magnitude between the candidate blinkers and non-blinkers, with non-blinkers showing little or no AB and blinkers showing a substantially larger AB. Similar findings were obtained when SOA 100 was excluded from the analysis.

### Experiment 1B

**Figure 2:**
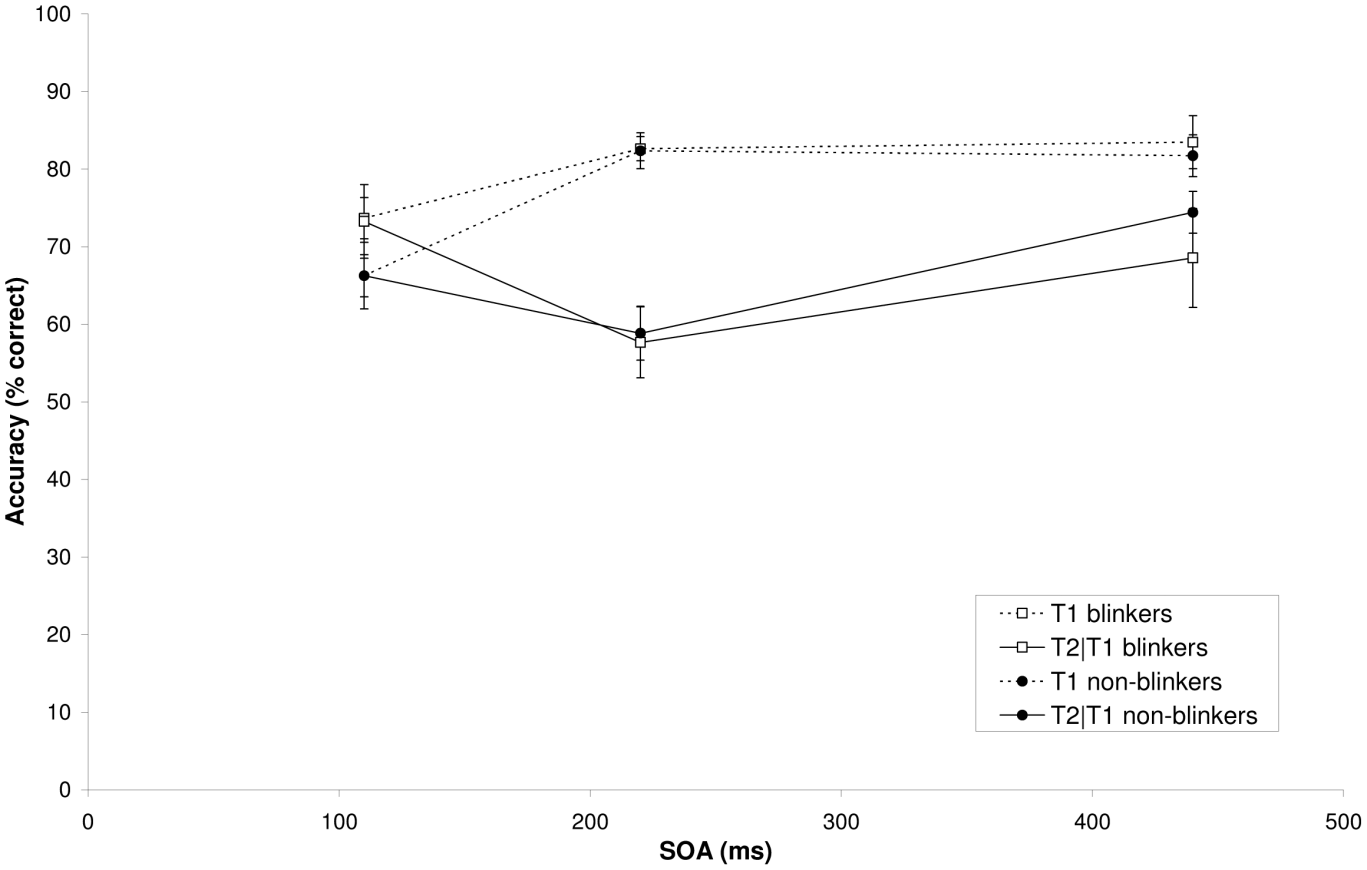
Target accuracy in Experiment 1B. Mean percentage correct report of T1 (dotted lines) and T2 given correct report of T1 (solid lines) as a function of SOA for blinkers (square symbols) and non-blinkers (circle symbols) in the picture AB task of Experiment 1B.

Figure 2 shows T1 performance in the picture AB task as a function of SOA for both groups. Mean T1 performance was 76.8% for the non-blinkers and 79.9% for the blinkers. An ANOVA on T1 performance with group (non-blinkers and blinkers) as between-subjects factor and SOA (110, 220, and 440 ms) as within-subjects factor revealed a significant effect of SOA, F(2, 44) = 17.09, MSE = 74.02, p<.001, η^2^_p_ = .44, reflecting worse performance at SOA 110 (lag 1). Neither a significant effect of group (p = .18) nor a Group×SOA interaction (p = .33) was found.

Figure 2 also shows T2 performance given that T1 was identified correctly, as a function of SOA for both groups. An ANOVA revealed a significant effect of SOA, F(2, 44) = 10.70, MSE = 115.98, p<.001, η^2^_p_ = .33. Neither a main effect of group (F<1) nor a Group×SOA interaction (p>.13) was found. Similar findings were obtained when SOA 110 was excluded from the analysis. In order to determine AB magnitudes for the picture AB task, we calculated T2|T1 relative to T1 for SOA 220. Mean AB magnitude for non-blinkers (28.0%) did not differ significantly from that of blinkers (30.4%; p = .72). Also when AB magnitude was calculated relative to T2 performance at the longest lag rather than T1 performance, AB magnitude did not differ significantly between non-blinkers (20.0%) and blinkers (13.2%; p = .43).

Finally, intra-individual stability of performance was checked on odd and even number trials for all participants. The Spearman-Brown prophecy coefficients were .48, .52, .21, and .32, for T1, T2|T1, AB magnitude relative to T1, and AB magnitude relative to T2 at SOA 440, respectively. The intra-individual stability was thus relatively low compared to what was found for Experiment 1A, or in previous AB studies [3], [5], [7], [10]. A likely explanation is that we only had a modest number of trials per condition (24), as well as a wide variety of pictures that differed in terms of identification difficulty. Moreover, whereas letter and digit stimuli in Experiment 1A were repeated many times throughout the experiment, each single stimulus in Experiment 1B was unique and was presented only once. Importantly, however, we found a substantial and statistically significant AB for both groups, which remained significant when either group was analyzed separately (ps<.01). Taken together, we conclude that blinkers and non-blinkers showed a substantial and comparable AB in the picture AB task.

### General Discussion

An aspect of the AB that is often ignored is the presence of large individual differences in the magnitude of the effect. In the current study (Experiment 1A), we replicated the previously reported finding that some individuals, referred to as non-blinkers, show little or no AB [3], [4], [5], [7], [8]. In Experiment 1B, one group of non-blinkers and a group of blinkers (who do show a substantial AB) were tested using an AB task adapted from Potter and colleagues [11], containing pictures rather than the more commonly used alphanumeric stimuli, in order to test the generality of the non-blinkers' remarkable ability. Amongst a stream of natural scenes presented at a rate of ∼9/s, two pictures (e.g., ‘bicycle’ and ‘airplane’) had to be identified that belonged to a superordinate category of objects (e.g., ‘vehicles’) that was specified at the start of each trial. A sizeable AB effect was observed for the blinkers, replicating recent findings reported by Potter et al. [11]. Importantly, however, an AB of similar magnitude was induced in the non-blinkers.

What factors may account for the finding that the non-blinkers blinked in the picture AB task? A first explanation that comes to mind is that the picture task was more demanding than the alphanumeric AB task, in a number of ways. Firstly, pictures are evidently more complex stimuli than letters and digits. In addition, each presented picture was unique and novel to the participants, whereas the letters and digits are highly familiar, overlearned stimuli, were repeatedly presented, and belonged to a limited stimulus set from only two stimulus categories (letters and digits). In contrast, the target pictures were defined at a high conceptual level, and a wide range of categories (32) and exemplars (2–8) was used. Moreover, participants were informed about the superordinate picture category on a given trial less than a second before the pictures appeared. Finally, it may have been more difficult to detect a target picture amongst other pictures than to detect a target letter amongst distractor digits.

However, mean T1 performance for the 24 selected participants was generally high, and only slightly lower in the picture task (78.3%) than in the alphanumeric task (84.6%), F(1, 22) = 19.40, MSE = 24.03, p<.001, η^2^_p_ = .47. Neither a significant effect of group (F<1), nor a significant Group×Task interaction (p = .14) was found, suggesting that the slightly increased task difficulty was similar for blinkers and non-blinkers. Given that an increase in task difficulty can lead to an increase in AB magnitude, the slightly lower T1 performance may thus be a potential explanation for the non-blinkers' blink in the picture task. However, this explanation is rendered somewhat implausible by the fact that the slight increase in task difficulty did not increase the size of the blinkers' AB. In fact, the blinkers' AB magnitude was significantly smaller in the picture task than in the alphanumeric task, t(11) = 2.85, SE = 4.95, p = .016.

An explanation for non-blinkers' problem with pictures may lie in the way that alphanumeric and picture stimuli are processed. It has been hypothesized that perceptual features of any stimulus are perceived in parallel early in processing, permitting detection of its category. Additional serial processing is subsequently required to bind those features to a specific object, so that it can be reported [12].

Indeed, there is electrophysiological evidence suggesting that global features and the category of alphanumeric stimuli are detected about 200 ms after presentation, and that full identification (including local features) follows about 50 ms later [13], [14]. In addition, it is known that category information can influence visual selection at an early stage in the processing pathway [15].

In the case of pictures, Evans and Treisman [12] have suggested that picture detection is similarly based on parallel processing of one or more features (e.g., “beaks”, “claws”, “fur”, and “eyes”) that are characteristic of the target category (e.g., “animals”), subsequently followed by the feature binding stage that leads to full identification. According to Evans and Treisman [12], the features of only one object can be bound at a time, thus providing a potential explanation for the AB (also see [16]).

However, Potter et al. [11] argued that if this were the case, no lag-1 sparing should occur when two unfamiliar pictures (T1 and T2) are presented in immediate succession (at lag 1). Using a similar paradigm to that used in the present study, they found substantial lag-1 sparing, as did we in Experiment 1B. This suggests that the specific object in a novel picture can be identified within about 100 ms, before or at the same time that it is categorized as a target, allowing an immediately following target to be identified as well. Unlike the category of a letter or a digit, the category of an object in a novel picture is unlikely to be detected before the object's specific identity.

It is thus possible that non-blinkers perform target selection in an alphanumeric AB task within an early processing stage, based for instance on alphanumeric category information [8], [15], [17], and that further processing of items is mostly restricted to targets only. In other words, non-blinkers do not blink in alphanumeric tasks because they have adopted an efficient strategy to separate targets and distractors at a much earlier stage of processing than the blinkers do. In contrast, pictures do not allow non-blinkers to use the same shortcut, leading to an AB of the usual magnitude in the picture AB task.

To conclude, the fact that non-blinkers blinked in a picture AB task but not in an alphanumeric AB task is unlikely to be due to differences in task difficulty. It is more likely that in the alphanumeric task non-blinkers take advantage of overlearned category-level features to select targets prior to full identification, allowing them to ignore distractors and avoid an AB [8]. Experiments are currently under way to test these possibilities.
